# Microbial Corrosion of Copper Under Conditions Simulating Deep Radioactive Waste Disposal

**DOI:** 10.3390/biology13121086

**Published:** 2024-12-23

**Authors:** Elena Abramova, Natalia Shapagina, Grigoriy Artemiev, Alexey Safonov

**Affiliations:** Frumkin Institute of Physical Chemistry and Electrochemistry, Russian Academy of Sciences, 119071 Moscow, Russia; gorchicta246@mail.ru (E.A.); fuchsia32@bk.ru (N.S.); artemyev56@gmail.com (G.A.)

**Keywords:** deep biosphere, geological repository for radioactive waste, copper biocorrosion, sulfate-reducing bacteria, copper toxicity

## Abstract

This paper presents the results of microbial corrosion tests on M0-grade copper under conditions simulating a geological repository for radioactive waste at the Yeniseisky site (Krasnoyarsk Krai, Russia). The work used a microbial community sampled from a depth of 450 m and stimulated with glucose, hydrogen and sulfate under anaerobic conditions. It was shown that the maximum corrosion rate, reaching 9.8 µm/y, was achieved with the addition of sulfate (1 g/L) with the participation of microorganisms from the families *Desulfomicrobiaceae*, *Desulfovibrionaceae* and *Desulfuromonadaceae*. It was noted that the most important factor leading to copper corrosion was the accumulation of hydrogen sulfide during the activation of sulfate-reducing microorganisms of the genera *Desulfomicrobium*, *Desulfovibrio* and *Desulfuromonas.* During the development of the microbial community under these conditions, the content of copper can have a significant toxic effect at a concentration of more than 250 mg/L.

## Simple Summary

Microbial communities living at a depth of 450 m in a granitoid massif (Krasnoyarsk Krai, Russia), potentially suitable for the construction of a radioactive waste storage facility, were studied. It has been established that their activation by organic matter, molecular hydrogen, can cause copper corrosion, which is significantly accelerated in the presence of sulfate ions. Microbial biofilms including sulfate-reducing and fermentative bacteria are resistant to copper ions and can reduce their toxicity during the formation of sulfide minerals.

## 1. Introduction

The disposal of radioactive waste (RW) in deep geological formations is currently regarded as the most reliable and safe way to isolate it from the environment [1]. This approach involves the use of a system of engineered safety barriers: metal containers to enclose radioactive materials, clay and cement materials to prevent moisture ingress and contact with the geological environment [2]. Due to the fact that one of the most important components of the security system is a metal container, countries that consider geological disposal of nuclear waste are concerned about the container’s stability and behaviour in a geological repository [3]. The Swedish and Finnish KBS-3 concept has chosen copper as the container material due to its comparatively high chemical resistance in the environment, unlike steel [4,5]. The corrosion of copper in storage facilities is characterised not by continuous (general) corrosion, but by localised corrosion, in particular in the presence of hydrogen [6], chloride [7], hydrocarbonate [8] and sulfide [9], and as a result of microbial corrosion (MIC) [10,11,12,13].

MIC can be intensified by both aerobic and anaerobic microorganisms including Bacteria, algae, protozoa, diatom, archaeon and fungi [14]. The major role, according to numerous studies, in copper corrosion is attributed to sulfur-cycle bacteria: sulfate-reducing bacteria (SRB) [15,16], sulfur-oxidising bacteria (SOB) [5], iron and manganese [16]. In addition, microbial biofilms are known to play an important role in microbial corrosion of copper [17]. Biofilms can accumulate organic acids that are involved in the dissolution of corrosion products that passivate the copper surface. Furthermore, microorganisms in biofilms are more protected from stressors such as exposure to toxic dissolution products of copper ions and radionuclides [18]. Several researchers [15,19,20,21] have proposed a mechanism for microbial corrosion of copper based on the oxidation of copper by hydrogen sulfide, with the role of SRB also being reduced to a decrease in the local pH, an important factor controlling MIC. In this case, extracellular polymeric substances (EPS) are responsible for creating preferential cathodic sites and produced acidic metabolites which may be associated with high copper by-product release [22,23]. The review [24] explains that there is no complete understanding of the mechanism of copper corrosion due to its complexity: it is complex and depends on microbial diversity.

Currently, the Yeniseisky rock massif (Krasnoyarsk Krai) is being considered for disposal of solidified radioactive waste after SNF reprocessing in the Russian Federation [25,26]. So far, the following materials are under consideration for the container in the storage concept: steels of various grades (mild and alloyed), cast irons and copper [27,28]. Copper is also being considered as an anti-corrosion coating material for stainless steel containers.

According to the data of the chemical composition of groundwater sampled in the Yeniseisky massif, carbonate and chloride corrosion may be the most important factors in terms of chemical corrosion of copper. The results given in our previous publications [29] have revealed that the clay materials to be used in the deep disposal facility for radioactive waste, rocks and pore waters sampled at different depths around the Yeniseisky site contain microorganisms that promote corrosion of mild steel, producing hydrogen sulfide, methane and hydrogen. The objective of this work was to evaluate the role of underground microflora of the Yeniseisky site (Krasnoyarsk Krai) leading to corrosion of copper as one of the container materials under consideration, as well as to search for the most important biogenic factors of corrosion.

## 2. Materials and Methods

Rectangular plates of technical copper grade M0 (Cu—99.95 wt. %, O—0.05 wt. %) of size 10 × 15 × 1 mm were used in this work. Before the studies, the samples were cleaned in an ultrasonic bath ‘Sapphire—0.8 TC’ (Moscow, Russia) in a mixture of C_2_H_5_OH:C_7_H_8_ = 1:1 for 25 min. The plate samples were suspended in 50 mL liquid medium, after which the vials were hermetically sealed and evacuated, and then argon (100%, extra pure) was added to the gas phase to create an anaerobic environment. The volume of the gas phase was 50 mL. The durations of the corrosion tests were 3, 10, 20, 45, 90 and 120 days.

The liquid phase was sampled from well R-8 (sample NW) of the Yeniseisky site (Krasnoyarsk region, Russia), where the future deep disposal facility for radioactive waste is expected to be constructed, with a sampling depth of 450 m. The sample was collected under aseptic conditions using a pre-sterilised sampler and placed into hermetically sealed sterile vials. The samples were fixed with ethanol to 30 vol% for molecular biological analysis. As for chemical analysis and laboratory experiments, samples were poured under the bottle top and stored at 4 °C. When analysed, the following ion concentrations (mg/L) were found in the sample: Mg^2+^—11.1, Ca^2+^—11.3, K^+^—2.1, SO_4_^2−^—1.51, Cl^−^—8.9, HCO_3_^−^—173.8, NO_3_^−^—3.0, CO_3_^2−^—10.5, Na^+^—52.6, pH 7.8.

During corrosion experiments, a sterile (autoclave sterilisation at 1.1 atm for 60 min) groundwater sample (NWS) was used as a control. Microbial stimulation was performed by the addition of hydrogen in the gas phase (sample NW-H), glucose (1 g/L) (sample NW-G) and a mixture of glucose (1 g/L) and sulfate ions (Na_2_SO_4_ 1 g/L) (sample NW-GS). All reagents were of high grade (e.g., >98%) (Merc).

To evaluate the role of individual microbial corrosion factors, the components NaHCO_3_—1 g/L (sample NWS-C), Na_2_S—1 g/L (sample NWS-Sd), Na_2_S—1 g/L and NaHCO_3_—1 g/L (sample NWS-CSd), were added to sterile groundwater, as listed in Table 1.

All experiments were performed in three replicates.

Microbial diversity. DNA was isolated using the ZymoBIOMICS™ DNA Miniprep Kit (Zymo Research, Tustin, CA, USA) according to the manufacturer’s instructions. Variable regions of the 16S rRNA gene in the V3-V4 region were selected for amplification in the preparation of libraries. DNA amplification was carried out by real-time PCR on CFX96 Touch (Bio-Rad, Hercules, CA, USA) with the qPCR mix-HS SYBR reaction mixture (Evrogen, Moscow, Russia). While preparing libraries for amplification, variable parts of the V3-V4 region of the 16S rRNA genes were selected: for amplification of the V3-V4 region, the degenerate primers For341 (5′-CCTACGGGNBGCASCAG-3′) and Rev806 (5′-GGACTACHVGGGTWTCTAAT-3′) were used. For amplification of the V4 region, the degenerate primers For515 (5′-GTGBCAGCMGCCGCGGTAA-3′) and Rev806 (5′-GGACTACHVGGGTWTCTAAT-3′) were used. Amplification was performed by real-time PCR on a CFX96 Touch (Bio-Rad, USA) using the qPCR mixHS SYBR (Evrogen, Moscow, Russia). Denaturation, primer annealing and chain elongation for regions V3-V4 were performed at 96, 54 and 72 °C, respectively. And the steps for region V4 were performed at 96, 58 and 72 °C, respectively. The purification of the desired product from each batch was carried out using Agencourt AMPure XP magnetic particles (Beckman Coulter, Brea, CA, USA). In addition, high-throughput sequencing was performed using a MiSeq system (Illumina, San-Diego, CA, USA) using a reagent kit (MiSeq Kit v2, 500 cycles, Illumina, USA).

Ion concentrations in water sample were determined using a Capel-205 capillary electrophoresis system (Lumex, Sankt-Peterburg, Russia) of the latest generation. Identification and quantification of analysed cations and anions were carried out by indirect method by measuring UV-absorption at 254 nm. Electrophoresis was carried out in untreated fused silica capillaries, 60 cm long (effective length—50 cm) and with a 75 µm inner diameter. The capillary was incubated at 20 °C with an applied voltage of +13 kV for cations or −17 kV for anions.

The biofouling and toxic effect of copper ions was assessed using the MTT method on the samples surface and in the liquid phase [28]. MTT test measures the cell respiratory activity by the optical density of reduced formazan dissolved in dimethyl sulfoxide, measured at a wavelength of 540 nm. Values of reduced formazan optical density (respiratory activity) are normalised to 1 cm^2^.

In order to study the toxic effect, different concentrations of copper ions in the form of copper sulfate were added to the liquid phase.

The corrosion rate was estimated by the gravimetric method. To remove corrosion products, 1.5 M H_2_SO_4_ solution (3 min at room temperature) was used.

The corrosion rate in µm/year was calculated by Formula (1):

V_cor_ = Δm × 8760/S × t × ρ (1)

where Δm is the average mass difference of samples before/after testing, [g], S is the surface area of the metal, [m^2^]; t is the testing time, [h], ρ—is the density of the metal, [g/cm^3^].

Changes in surface morphology were assessed using a Carton SPZT50 optical microscope (manufactured in Carton optical, Kanagawa, Japan) with a magnification of 2×, equipped with a digital camera DCM510. The camera resolution in pixels was 2048 × 1536. The area of the observed image was 1 cm^2^. The duration of the experiments was 24 h. The imaging periodicity was 5 min. After the corrosion tests, the corrosion lesions on the surface were evaluated according to the criteria of ASTM D 610-01 [30].

Electron microscopy was carried out using a TESCAN MIRA3 FEG-SEM (Warrendale, Pensilvania, PA, USA) scanning electron microscope from the Joint Use Center, Vernadsky Institute of Geochemistry and Analytical Chemistry, Russian Academy of Sciences. The samples were taken in two modes, SE and BSE, at a voltage of 20 kV.

Volatile fatty acids (VFAs) were determined using a Crystal 5000.2 gas chromatograph (Chromatec, Moscow, Russia) equipped with a flame ionisation detector and a capillary column ZB-WAXplus 30 m × 0.25 mm × 0.35 µm (Phenomenex, Torrance, CA, USA). The column was operated with temperature programming from 100 to 180 °C with a ramp rate of 10 °C/min. The gas carrier was nitrogen.

## 3. Results

### 3.1. Microbial Fouling of Samples and Changes in Microbial Community Composition

#### 3.1.1. Microbial Fouling of Samples

**Figure 1:**
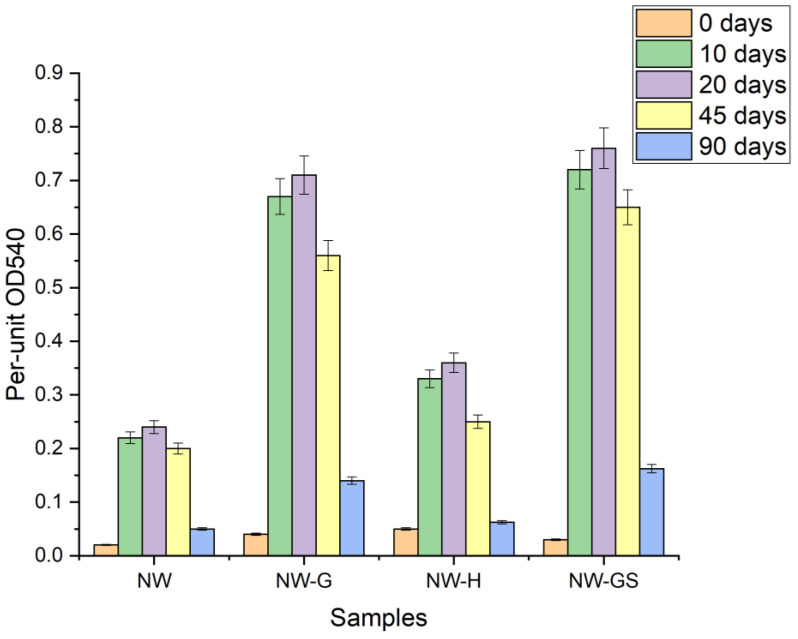
Respiratory activity of biofilms on the surface of 1 cm^2^ of samples at 0, 10, 20 and 45 days.

Evaluation of microbial biofouling on the surface of copper samples (Figure 1) showed that maximum biofilm growth was observed for all samples by 20 days. The maximum values of biofilm respiratory activity were found on the copper surface when the microbial community was stimulated with glucose and a mixture of glucose and sulfate. It is important to note that without stimulation the intensity of microbial respiration was 3 ÷ 4 times lower than in the experiments with glucose stimulation. Biofouling efficiency under hydrogen stimulation was lower than under glucose stimulation by 2 times on average. A significant decrease in microbial activity on the 90th day occurred after a single stimulation of the microbial community with organic matter.

#### 3.1.2. Volatile Acid Formation

**Figure 2:**
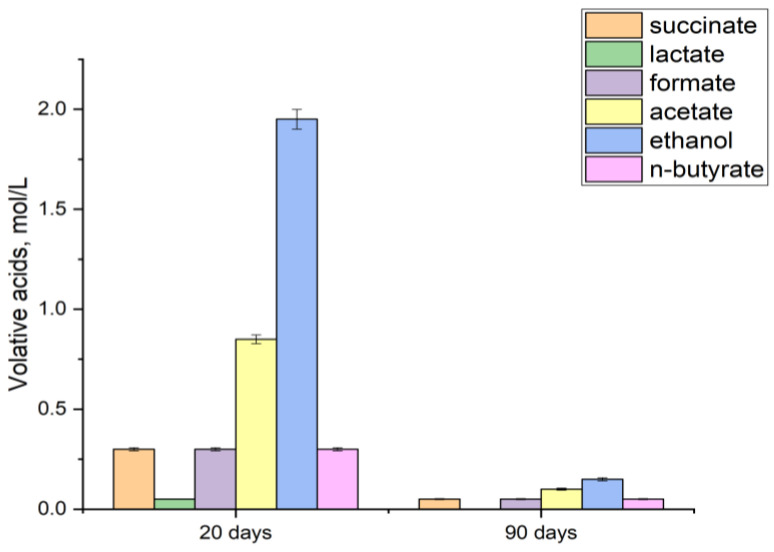
Volatile acid content on the 20th day of the experiment under glucose stimulation at 20 and 90 days.

It was found that on the 20th day of the experiment (Figure 2), the accumulation of succinate, formiate and n-butyrate in the medium was detected at a concentration of 0.3 mol/L, lactate—0.05 mol/L, acetate—0.85 mol/L, ethanol—1.95 mol/L. It is important to note that by day 30 the concentration of organic metabolites in the solution had decreased by an average of 80–90% due to their consumption as a result of microbial succession. It can be assumed that at the initial stage there was the development of processes of anaerobic fermentation of glucose and further consumption of fermentation products by anerobic microorganisms under conditions of deficiency of organic matter. Thus, the role of organic metabolites in copper corrosion can only be performed in the early stages of community development (up to 20–30 days).

#### 3.1.3. Microbial Diversity of Groundwater Samples

**Figure 3:**
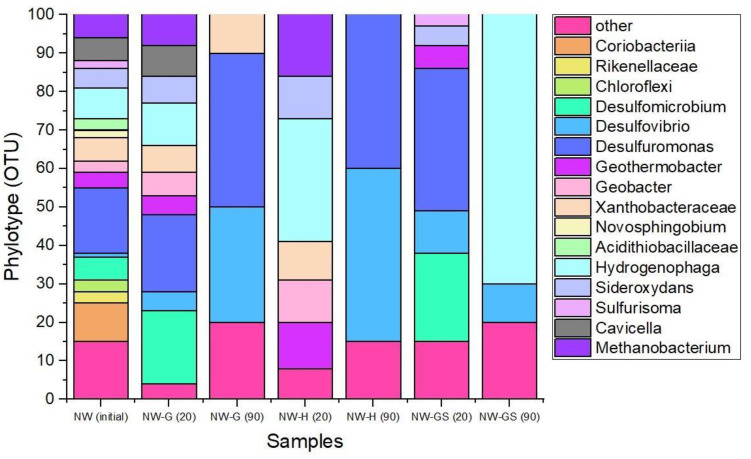
Initial microbial diversity (OTU, %) of experiment samples, and at 20 and 90 days.

In the sample (NW) of groundwater from the well (Figure 3), the dominant groups were species of *Hydrogenophaga*, *Desulfomicrobium*, *Desulfovibrio*, *Desulfuromonas*, *Geothermobacter*, *Geobacter*, *Smithella*, *Methanobacterium*, *g.Cavicella*, *g.Coriobacteriia*.

In the NW-G sample with glucose addition after 20 days of the experiment, the species of phylum *Desulfuromonas*, *Hydrogenophaga*, *Methanobacterium* and the family *Xanthobacteraceae*, possessing predominantly the fermentative type of metabolism under anaerobic conditions, were dominant.

In the NW-H sample with hydrogen addition, the dominance of bacteria of the taxa *Geobacter*, *Xanthobacteraceae*, *Geothermobacter*, *Hydrogenophaga* and *Methanobacterium*, capable of iron reduction, was observed. A number of species can reduce sulfates in assimilation processes and digest organic compounds, e.g., metabolic products.

In NW-GS sample with addition of glucose and sulfate ions at 45 days, the dominance of the taxa *Desulfomicrobium*, *Desulfovibrio*, *Desulfuromonas* and *Sideroxydans*, capable of assimilatory sulfate reduction, iron reduction and glucose digestion, was found.

By day 90 of the experiment, a significant decrease in microbial diversity was observed in all samples. In the NW-H and NW-G sample, the dominant groups were bacteria of the sulfur cycle *Desulfovibrio*, *Desulfuromonas* and *Desulfomicrobium. Hydrogenophaga* dominated in the NW-GS sample representatives of the taxon.

### 3.2. Evaluation of Corrosion Damage of Samples

#### 3.2.1. Optical Microscopy and Visual Analysis of Samples

**Figure 4:**
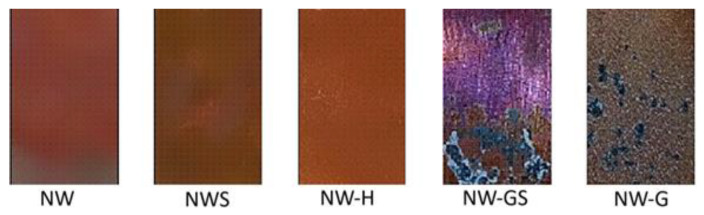
Surface of copper plates after incubation in different media for 45 days.

According to visual evaluation of the samples (Figure 4) pulled out of the solutions after 45 days, the maximum corrosion lesions were characteristic of the sample with the addition of glucose and sulfate with the total area of corrosion lesions—82 ± 4% of the total surface area of the plates. In the case of glucose stimulation, localised corrosion foci with a total area of 7% were observed on the surface. In the case of the sterile sample (NWS), the control sample without stimulation (NW) and the sample with hydrogen stimulation (NW-H), general surface corrosion was observed, while local foci were not observed.

**Figure 5:**
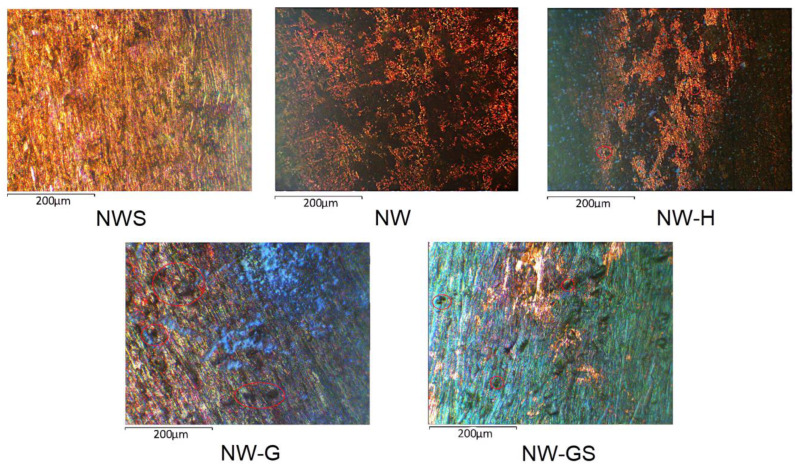
Microphotographs of M0 surface after incubation of samples in different media for 45 days.

In Figure 5, the results of the corrosion tests of copper specimens aged for 45 days in different media are presented.

In the sterile natural water (Sample NWS) as well as in the natural water conditions (Sample NW), there were no local defects on the copper surface. In these media, the entire metal surface underwent uniform etching. In experiments with activated microbiota (NW-G and NW-H), localised foci of metal dissolution were recorded on the copper surface. The average number of defects (N_avg._) per 1 mm^2^ in NW-H medium was three defects, and the average diameter (d_avg._) of defects was 10.3 μm. In the case of sample NW-G, N_avg._ = four defects with d_avg._ = 10.6 μm. In the presence of SRB with the addition of sulfate ions (sample NW-GS), a greater number of local defects were found (the average number of defects per 1 mm^2^ of visible surface was six, with a diameter of 12.7 μm).

#### 3.2.2. Evaluation of Corrosion Rates

**Figure 6:**
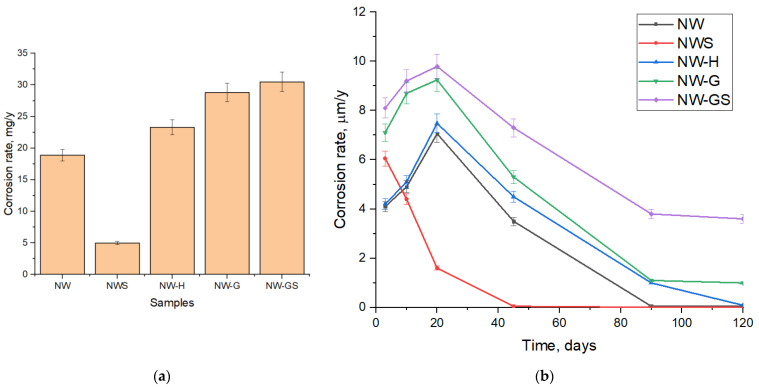
Corrosion rate of copper samples: (**a**) maximum corrosion rate, mg/yr, at 20 days; (**b**) corrosion rate kinetics.

According to the data of gravimetric analysis (Figure 6), it was found that the maximum corrosion rate reaching 9.8 μm/year was characteristic of the experiment in which glucose and sulfate stimulation was carried out on the 20th day of the experiment. Importantly, the corrosion rate in the sterile sample reached 1.8 μm/year and was not significantly different from the sample with an unstimulated microbial community. When stimulated with hydrogen, the corrosion rate increased 1.3-fold and reached 7.5 µm/yr. Stimulation of an organotrophic microbial community resulted in a 1.5-fold increase in the corrosion rate.

The results of assessing the corrosion rate at different time intervals (Figure 6b) showed that in biological experiments the maximum was observed on the 20 ÷ 30th day, which coincided with the maximum of surface biofouling. In the abiotic experiment, the maximum corrosion rate was reached by the 3rd day, after which it gradually decreased and reached zero values by the 30th day. Moreover, in biotic experiments, the achievement of pseudo-equilibrium corrosion rates was observed by 90 days.

#### 3.2.3. Evaluation of Corrosion Product Composition

**Figure 7:**
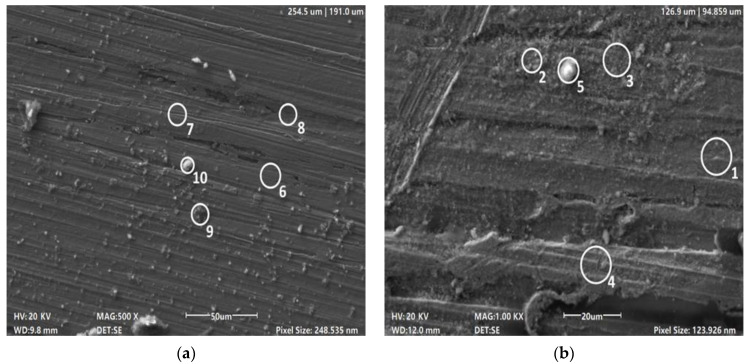
Surface micrographs of NW-G copper (**a**) and NW-GS (**b**) on day 45.

After the development of SRB in the NW-GS experiment on day 45 (Figure 7 and Table 2), on the copper surface were detected copper oxide phases (point 2) and microbial biofilms with the inclusion of copper and iron sulfide phases (points 3, 4), as well as carbonate phases with copper, calcium and sodium, possibly containing chalconanthronite, a product of carbonate corrosion of copper (point 5). The spectrum of point 1 characterises the surface composition of weakly corroded copper, but it has 2.5 wt% of oxygen and 1.8 wt% of sulfur, which may indicate the ongoing process of sulfide corrosion. In general, it is possible to explain the increased content of sulfur in all investigated points, in comparison with the experiment without addition of sulfates, which can speak about the important contribution of sulfide corrosion in conditions of development of SRB.

Following the development of organotrophic microorganisms under glucose stimulation (NW-G experiment on day 45), copper oxide films (point 7) containing sodium, calcium and carbon were found on the copper sample, which can be explained by the formation of chalconanthronite. Traces of chlorine may also indirectly indicate chloride-mediated copper corrosion processes. The formation of chalconantronite phase can also be assumed on the basis of the composition of point 10, which includes copper, sodium, carbon and calcium. Microbial biofilms (points 8 and 9) were found on the surface of the sample; the biogenic nature of these phases is indicated by the presence of biophilic elements Cl, K, Na, Ca and Fe. Only sulfur was present in the biofilms, probably in sulfide form, which indicates in this case the important role of biofilms in copper sulfide corrosion. The spectrum of Point 6 characterises the composition of the weakly corroded copper plate and has a small amount of carbon in its composition, possibly as part of carbonates of microbial origin. Small amounts of sodium and calcium may also indicate the process of carbonate corrosion of copper with the formation of chalconanthronite.

### 3.3. Evaluation of Copper Toxicity

A slight decrease in the respiratory activity of microorganisms (Table 3) with copper plates was found to occur in planktonic culture on the 20th day. A maximised decrease up to two times was found in the sample with natural water without stimulation. In the sample with glucose and glucose-sulfate stimulation, a less significant decrease in the respiratory activity of planktonic microorganisms was found, of 1.4 and 1.2 times, respectively. This is probably due to the development of microbial biofilms and the accumulation of copper ions in the composition of organic phases.

**Figure 8:**
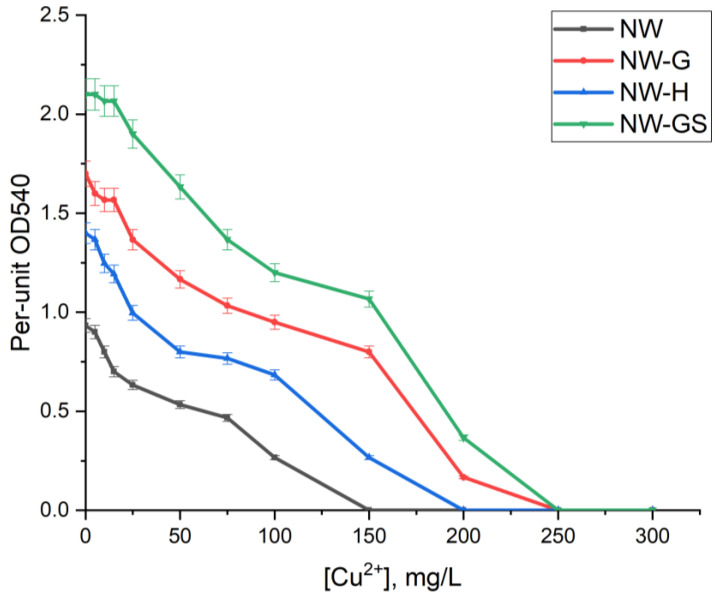
Respiratory activity of planktonic microorganisms at different copper concentrations.

The toxicity of copper ions to the microbial community under different variants of its stimulation was assessed in a model experiment (Figure 8). When copper ions were added, the greatest effect of copper ions was found for communities in water without stimulation and with hydrogen stimulation. In these cases, copper ion concentrations starting at 10 mg/L for NW and 20 mg/L for NW-H are significant. In the case of experiments with glucose stimulation, the decrease in respiratory activity started from 30 ÷ 40 mg/L. The calculated lethal dose (LD100) and median lethal dose (LD50) values for the planktonic and attached community are given in Table 4. Maximum LD50 values for the planktonic culture were obtained at copper concentrations of 100 and 150 mg/L for glucose stimulation experiments. For the culture in biofilm, the semi-lethal and lethal doses for the experiments with glucose stimulation increased one and a half times, up to values of 200 and 350 ÷ 400 mg/L of copper. For the experiment without stimulation, no significant differences in the values of lethal and semi-lethal doses were observed compared to the planktonic culture.

## 4. Discussion

Table 5 provides a summary table showing the relationship between biofouling, microbial diversity and corrosion rates on day 20 of the experiment during the peak of microbial activity.

Evaluation of microbial community diversity under different types of stimulation showed that the addition of glucose leads to the development of microorganisms mainly with the fermentation type of metabolism (*Xanthobacteraceae*, *Hydrogenophaga*, *Cavicella*), which leads to an increase in the corrosion rate compared to the abiotic control by one and a half times and the appearance of localised corrosion foci (average number of four per 1 mm^2^). An important role of *Desulfuromonas* in copper corrosion was noted in [31].

The species of the genus *Cavicella* are organotrophs or chemoorganotrophs and can reduce nitrates, sulfates and iron. The species of *Coriobacteriia* mostly have a fermentative type of metabolism, which contributes to the formation of organic acids and carbon dioxide [32]. The addition of sulfate together with glucose leads to the stimulation of sulfate-reducing microorganisms (*Desulfomicrobium*, *Desulfovibrio*, *Desulfuromonas* [33]), which are known for their role in copper corrosion and are capable of sulfate reduction [34], in some cases using hydrogen as an electron donor [35]. Under these conditions, a twofold increase in the corrosion rate and the appearance of localised corrosion centres were observed. It is important to note that in the latter case, the character of corrosion lesions is markedly different from the other conditions; local foci of corrosion were found (the average number of defects per 1 mm^2^ of surface was six) and the average diameter increased to 12.7 μm. Upon stimulation with hydrogen, in spite of a not-so-significant increase in corrosion rate, we also observed the appearance of separate corrosion centres with total number of three per 1 mm^2^. Probably in this case, there was a hydrogen mechanism of steel corrosion.

### 4.1. Biofilm Formation and Its “Dual Effects”

In the case of glucose stimulation, both with and without sulfate, the most active biofilm development was observed on the surface of the samples, formed mainly by organotrophic bacteria. In the study [36], 75% of the of biofilm bacterial community on copper was composed of bacteria of the family *Xanthobacteraceae*. The majority of organisms, including species of *Xanthobacteraceae* and *Desulfovibrio*, found in the community under different cultivation conditions are able to form biofilms on copper [36].

Within biofilms, when stimulated with glucose without sulfate addition, methane- and sulfur-oxidising bacteria, found in most samples, may develop, which may contribute to the corrosion reaction under different conditions of oxidation-reduction potential [37]. When organotrophic microorganisms (*Xanthobacteraceae*) develop, sulfate reduction may occur through assimilative processes. In the case of a deep disposal facility for radioactive waste, organic matter may be introduced by the contact of copper with clay barriers [38,39]. Stimulation of microbial processes by molecular hydrogen in conditions of deep disposal of radioactive waste can occur due to its terrogenic [40] or radiolytic origin [41]. Based on the data obtained in this study, the most important role in the microbial processes of copper corrosion is played by sulfate ions, since the process of sulfate reduction can occur in lithotrophic conditions, microorganisms found in the conditions of deep disposal of radioactive waste [42].

In studies [38,43], *Geothermobacter*, *Hydrogenophaga* and *Geobacter* were found in biofilms on the surface of copper. The species of the genus *Hydrogenophaga* are known for their ability in methane formation [44] and fermentation. A number of species of this family use molecular hydrogen as an electron donor [45].

The species of the genus *Methanobacterium* are predominantly anaerobic bacteria that reduce methane [46].

### 4.2. Dominant Role of Sulfate-Reducing Bacteria (SRB) in Copper Corrosion

Numerous studies on the microbial corrosion of copper in the conditions of deep disposal of radioactive waste have established that microbial corrosion has several mechanisms, including the effect of biogenic sulfide ions, carbonate and organic acids, which are most active in the microbial biofilm. It was shown in [38] that copper cathodic depolarisation can occur within biofilms, which is accelerated by SRB or methanogenic bacteria, with both organotrophic microorganisms consuming organic matter of biofilms and H_2_—using bacteria playing an important role. Based on our studies, it was found that biofouling of the surface of copper plates was observed in all experiments, including the experiment without the introduction of growth stimulants, under the conditions of the microbial community characteristic of the Yeniseisky site. The intensity of biofouling increased more than twofold when hydrogen was added.

In the study [47], it was proposed that the key initiating reaction is abiotic oxidation of copper, which then, interacting with metabolic products such as oxalate, forms poorly soluble precipitates; in addition, microbial formation of hydrogen sulfide leads to its oxidation of the CuO surface with the release of hydrogen, consumed by lithotrophic microorganisms with the formation of secondary copper sulfide phases. According to the data of electron microscopy on the surface of copper M0 samples, we found areas with accumulation of corrosion products in the form of oxides, sulfides and carbonates, which can potentially indicate the processes of sulfide and carbonate corrosion of copper in biofilm. Following the literature data [48,49], it can be assumed that the protective passive film on the copper surface is “two-layered”: the first layer (Cu_2_O) is dense and firmly bound to the metal surface, while the second layer (CuO) has a porous structure. As a result of structural differences, electrical resistance increases at the dense/porous layer interface, which leads to further oxidation of the porous corrosion products layer. The existence of Cu_2_O oxide coated with CuO/Cu(OH)_2_ on the copper surface was confirmed by X-ray photoelectron spectroscopy and secondary ion mass spectroscopy [50,51]. Over time, the compound Cu(OH)_2_ is converted to CuO, which is the final, stable product of the second step of copper oxidation [52].

The identification of sulfide is most likely the result of microbial influence. Under standard abiotic conditions, sulfide formation by sulfate reduction is difficult, and thus the presence of sulfide is related to SRB activity. The authors of [53] observed the transformation from copper oxides to sulfides through a chemical substitution reaction between oxide and sulfide: Cu_2_O + SH^−^ → Cu_2_S + OH^−^. The authors of [54] suggested that the resulting metabolite HS^−^ of H_2_S produced by SRB is able to diffuse to the copper surface and react with it to form Cu_2_S by the reaction 2Cu + HS^−^ + H^+^ → Cu_2_S + H_2_. As a result, on the Cu surface the biofilm is forming. On one hand, the presence of biofilm on the copper surface leads to a slowdown of corrosion processes, as it acts as a barrier separating the metal from the aggressive environment. However, on the other hand, take into consideration the fact that the sulfide film is very fragile and can crack even at insignificant variations of potential in comparison with the oxide film. Consequently, there is an occurence of bare metal areas that dissolve under the influence of hydrogen and sulfur ions, which in turn leads to the regeneration of the copper sulfide film. This cyclical process leads to a fairly rapid appearance of local corrosion damage in the form of pitting. The presence of residual dissolved oxygen in the solution leads to an even greater increase in the rate of local corrosion. A similar mechanism of localised corrosion defects occurs in the case of hydrogen carbonate [55].

The corrosion of high-purity copper by SRB in a groundwater well or MX-80 clay was demonstrated in [56], where the formation of Cu_2_S was observed. The presence of acetate, lactate and sulfate was found to increase bacterial activity, resulting in the formation of corrosion products on copper discs inside compacted bentonite blocks in the absence of water flow resumption after one year of oxygen-free incubation [38]. The presence of the aqueous phase accelerated this process, and the formation of corrosive compounds was observed at an early stage of incubation (45 days) [37].

### 4.3. Synergistic Acceleration of Corrosion by Multiple Environmental Factors

**Figure 9:**
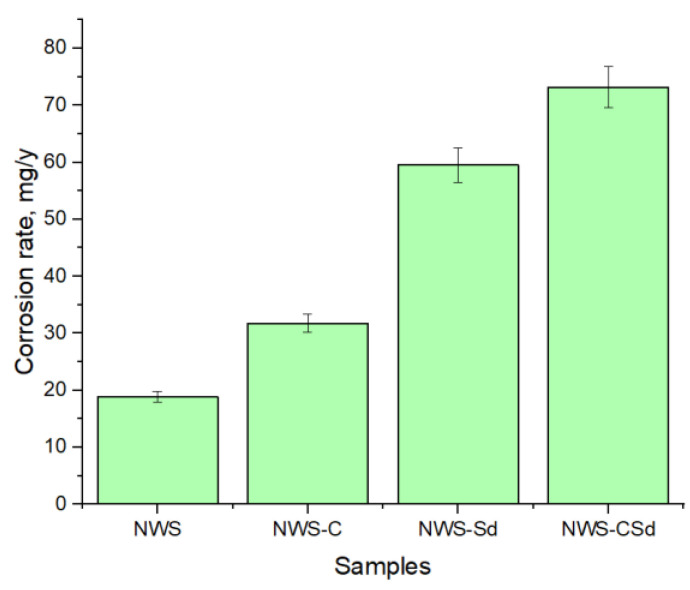
Corrosion rate of copper samples under sterile conditions in the presence of microbial corrosion components (NWS—sterile groundwater, NWS-C—with added carbonates, NWS-Sd—with added sulfides, NWS-CSd—with added carbonates and sulfides): a) maximum corrosion rate, mg/y, at three days.

To assess the role of sulfide and carbonate corrosion under conditions simulating the Yeniseisky deep disposal site for radioactive waste, model chemical experiments were carried out with samples in a sterilised groundwater sample (Figure 9). It was found that under abiotic conditions, the most important factor is the addition of sulfide, which increases the corrosion rate by 2.5 times, while the addition of hydrogen carbonate leads to an increase in the corrosion rate by 1.5 times. The combined effect of sulfide and hydrogen carbonate ions leads to an increase in the corrosion rate up to 73 mg/year.

The data on the evaluation of copper ion toxicity obtained on the example of a microbial community sampled at the depth of the Yeniseisky site agree with a number of works. In the study [57], it was established that copper ions have an inhibitory effect on sulfate reducers at concentrations starting from 20 mg/L. At the same time, it is known that SRB have several mechanisms for copper detoxification, for example by releasing organic complexes [58]. In this case, resistant strains can actively develop at concentrations ranging from 1 to 20 mM Cu. The high stability of microorganisms in biofilm is explained by chelation of copper by polysaccharides [59] and precipitation in the form of sulfides, which leads to its localisation [60].

Therefore, biofilm formation plays an important role in microbial corrosion processes of copper. In biofilm, anaerobic sulfate-reducing and methanogenic bacteria develop, leading to the accumulation of metabolites and contributing to copper corrosion. It protects microorganisms from the influence of copper ions, due to the accumulation of copper organic and mineral phases.

### 4.4. Corrosion Mitigation and Antimicrobial Strategies in Deep Geological Disposal

The highest risk of microbial processes is in the areas of contact of copper with clay materials. The inflow of organic substances and biophilic elements from clays can lead to an increase in the rate of corrosion. The reduction of microbial activity in the conditions of deep radioactive waste disposal can be reduced by introducing biocidal additives into clay materials. We have previously proposed effective biocidal additives for clays based on quaternary ammonium compounds, boric acid and guanidine (PHMG) [29], reducing sulfate-reduction rates by 5–6 times with “Amanate”, PHMG by 4–40 times and boric acid by 2 times [61].

## 5. Conclusions

In the conditions of the Yeniseisky radioactive waste deep disposal site there is a physiologically diverse microbial community, including fermenting and SRB, which in the conditions of ingress of sulfate ions, molecular hydrogen, is able to participate in the processes of copper corrosion. A significant increase in the rate of copper corrosion, reaching 9.8 μm/year, is achieved with the activity of SRB. The processes of anaerobic fermentation of organic matter, which may be contained in clay minerals, may also play an important role in the process of copper corrosion. However, the consumption of organic matter, in the conditions of underground storage, may occur at the first stages of microbial succession. At the same time, as it was shown in our study, it is the anaerobic fermentation processes that result in the maximum copper corrosion rates on the 20th day. These processes can initiate the development of SRB, using the products of their metabolism. Evaluation of the success of the microbial community in all experiments showed that in experiments with the addition of organic matter after the development of organotrophic bacteria on the 20th day, by the 90th day, mainly SRB remained. Biofilm formation plays an important role in the processes of microbial corrosion of copper. There is the development of anaerobic sulfate-reducing and methanogenic bacteria on the inside as well as an accumulation of metabolites and chemical processes of copper corrosion. It protects microorganisms from the influence of copper ions, due to the accumulation of copper organic and mineral phases.

Thus, the obtained data show the importance of taking into account microbial processes when substantiating the safety of the repository for deep disposal of radioactive waste at the Yeniseisky site. Even though relatively inert in geochemical conditions, the copper material of RW containers can be subjected to microbial and microbial-mediated corrosion, which can reduce its service life. To reduce these processes, it is necessary to use biocidal additives in clay materials. The results of this study provide initial data on the possible mechanisms of biogenic and chemical corrosion of copper containers. These will be further confirmed in long-term experiments in an underground research laboratory.

## Figures and Tables

**Table 1:** Description of experiments.

**1. Copper Biocorrosion by Microbial Community**			
**Conditions**	**Additions**	**Time, Days**	**Analyses**
– Groundwater sample R-8 (mg/L): Mg^2+^—11.1, Ca^2+^—11.3, K^+^—2.1, SO_4_^2−^—1.51, Cl^−^—8.9, HCO_3_^−^—173.8, NO_3_^−^—3.0, CO_3_^2−^—10.5, Na^+^—52.6, pH 7.8; – Plates of copper M0—10 × 15 × 1 mm; – Anaerobically (Ar, 100%), T = 20 °C	NW—control—groundwater NWS—sterile control NW-H—H_2_ as a gas phase NW-G—glucose (1 g/L) NW-GS—glucose (1 g/L) + Na_2_SO_4_ (1 g/L)	3, 10, 20, 45, 90, 120	– Microbial diversity assessment (16S rRNA) – Evaluation of microbial biofouling (MTT test) – Volatile acid content – Surface evaluation of copper samples (visual evaluation, optical ex situ microscopy, scanning electron microscopy) – Measurement of corrosion rate by gravimetric method
**2. The Effect of Microbial Components on Copper Corrosion**			
**Conditions**	**Additions**	**Time, Days**	**Analyses**
– Sterile groundwater R-8 – Plates of copper M0—10 × 15 × 1 mm – Anaerobically (Ar, 100%), T = 20 °C	NWS—sterile control NWS-C—NaHCO_3_ (1 g/L) NWS-Sd—Na_2_S (1 g/L) NWS-CSd—Na_2_S (1 g/L) + NaHCO_3_—(1 g/L)	3, 10, 20, 45, 90, 120	– Measurement of corrosion rate by gravimetric method

**Table 2:** Composition of points shown in Figure 6 according to EDX analysis data, wt%.

Point	Possible Phase Identification	C	O	Na	S	Cl	K	Ca	Fe	Cu
1	Copper	12.91	2.43	-	0.87	-	-		-	80.80
2	Copper oxide Cu_2_O	9.04	18.78	-	0.96	-	-	-	-	71.42
3	Biofilm with sulfides	25.12	44.32	0.87	9.88	0.34	0.77	0.28	1.22	39.96
4	Corrosion products with biofilm	27.84	48.21	0.69	7.89	0.29	0.81	0.24	0.75	36.06
5	Calcium carbonate possibly Chalconanthronite	22.19	54.09	6.78	1.87	-	-	12.38	-	8.65
6	Copper	1.68	3.90	0.12	-	0.20	-	-	-	89.21
7	Copper oxides	6.80	14.47	1.1	-	0.37	-	0.32	0.11	74.24
8	Biofilms corrosion products	29.22	34.36	0.18	3.09	0.55	0.13	0.93	0.15	65.28
9	Biofilms corrosion products	27.76	36.57	0.23	1.16	0.72	0.30	2.53	0.23	21.87
10	Calcium carbonate, possibly Chalconanthronite	49.68	26.66	3.81	-	-	-	4.00		6.93

**Table 3:** Respiratory activity of microorganisms in solution, at 20 days, relative units.

Conditions	Per-Unit OD540 in Solution	Per-Unit OD540 in Solution with Copper Plates	Microbial Activity Reduction Ratio
NW	0.93 ± 0.07	0.44 ± 0.04	2.1
NW-G	1.8 ± 0.14	1.3 ± 0.06	1.4
NW-H	1.3 ± 0.12	0.81 ± 0.05	1.6
NW-GS	2.1 ± 0.16	1.75 ± 0.13	1.2

**Table 4:** Lethal doses (LD100) and median lethal doses (LD50) to the microbial community during stimulation in planktonic culture and in biofilms.

System	Samples	LD50, mg/L	LD100, mg/L
Planktonic culture	NW	75	150
	NW-G	150	250
	NW-H	100	200
	NW-GS	150	250
Biofilms	NW	75	150
	NW-G	200	350
	NW-H	150	250
	NW-GS	200	400

**Table 5:** Generalised results of experiments on the 20th day.

Characteristics	NW	NWS	NW-H	NW-G	NW-GS
E_h_, mV	−75	−120	−100	−150	−180
pH	7.6	7.8	8.0	6.5	6.8
*Desulfomicrobium* (OTU), %	6	-	0	19	23
*Desulfovibrio* (OTU), %	1	-	0	5	11
*Desulfuromonas* (OTU), %	17	-	0	20	37
*Geothermobacter* (OTU), %	4	-	12	5	6
*Xanthobacteraceae* (OTU), %	6	-	10	7	0
*Hydrogenophaga* (OTU), %	8	-	32	11	0
*Methanobacterium* (OTU), %	6	-	16	8	0
Microbial activity, per-unit OD540	0.24 ± 0.01	-	0.36 ± 0.02	0.71 ± 0.04	0.76 ± 0.04
V cor, mg/y	18.9 ± 0.95	5.0 ± 0.25	23.3 ± 1.17	28.8 ± 1.44	30.5 ± 1.5
V cor, μm/y	7.06 ± 0.35	1.61 ± 0.08	7.48 ± 0.37	9.25 ± 0.46	9.8 ± 0.5

## Data Availability

Data are contained within the article.
